# The Coding and Small Non-coding Hippocampal Synaptic RNAome

**DOI:** 10.1007/s12035-021-02296-y

**Published:** 2021-02-10

**Authors:** Robert Epple, Dennis Krüger, Tea Berulava, Gerrit Brehm, Momchil Ninov, Rezaul Islam, Sarah Köster, Andre Fischer

**Affiliations:** 1 Department of Systems Medicine and Epigenetics, German Center for Neurodegenerative Diseases (DZNE), Von Siebold Str. 3a, 37075, Goettingen, Germany; 2 Bioinformatics Unit, German Center for Neurodegenerative Diseases (DZNE), Von Siebold Str. 3a, 37075, Goettingen, Germany; 3 Institute for X-Ray Physics, University of Goettingen, Goettingen, Germany; 4 Cluster of Excellence “Multiscale Bioimaging: from Molecular Machines to Networks of Excitable Cells” (MBExC), University of Goettingen, Goettingen, Germany; 5 Department of Neurobiology, Max-Planck Institute for Biophysical Chemistry, 37077 Göttingen, Germany; 6 Department of Psychiatry and Psychotherapy, University Medical Center Goettingen, Goettingen, Germany

**Keywords:** mRNA, microRNA, lncRNA, snoRNA, Synapse, Synaptosomes, Gene expression, RNA sequencing

## Abstract

Neurons are highly compartmentalized cells that depend on local protein synthesis. Messenger RNAs (mRNAs) have thus been detected in neuronal dendrites, and more recently in the pre- and postsynaptic compartments as well. Other RNA species such as microRNAs have also been described at synapses where they are believed to control mRNA availability for local translation. A combined dataset analyzing the synaptic coding and non-coding RNAome via next-generation sequencing approaches is, however, still lacking. Here, we isolate synaptosomes from the hippocampus of young wild-type mice and provide the coding and non-coding synaptic RNAome. These data are complemented by a novel approach for analyzing the synaptic RNAome from primary hippocampal neurons grown in microfluidic chambers. Our data show that synaptic microRNAs control almost the entire synaptic mRNAome, and we identified several hub microRNAs. By combining the *in vivo* synaptosomal data with our novel microfluidic chamber system, our findings also support the hypothesis that part of the synaptic microRNAome may be supplied to neurons via astrocytes. Moreover, the microfluidic system is suitable for studying the dynamics of the synaptic RNAome in response to stimulation. In conclusion, our data provide a valuable resource and point to several important targets for further research.

**Supplementary Information:**

The online version contains supplementary material available at 10.1007/s12035-021-02296-y.

## Introduction

Neurons are highly compartmentalized cells that form chemical synapses, and the plasticity of such synapses is a key process underlying cognitive function. In turn, loss of synaptic integrity and plasticity is an early event in neuropsychiatric and neurodegenerative diseases. Synapses are usually far away from the soma, which raises the question as to how neurons ensure the supply of synaptic proteins. Theoretical considerations and a substantial amount of data show that mRNAs coding for key synaptic proteins are transported along dendrites to synaptic compartments, where they are locally translated into proteins [1–5]. Hence, several studies have investigated the synaptic RNAome using different approaches. For example, early *in situ* hybridization experiments demonstrated the localization of specific mRNAs to synapses [6]. In addition, microarray and RNA-seq techniques were used to study the synapto-dendritic [7–9], synapto-neurosomal [10], and more recently, also the synaptosomal RNA pool of the mouse brain [11, 12]. However, compared with mRNAs, there is less known about the non-coding RNAome at synapses. The best known non-coding RNAs are microRNAs, which are 19–22 nucleotide-long RNA molecules that regulate protein homeostasis via binding to a target mRNA, thereby causing its degradation or inhibition of translation [13]. Several microRNAs were found to be implicated in synaptic plasticity, and these were identified at synapses where they were linked to the regulation of mRNA stability and availability for translation [14–17]. However, a combined analysis of the synaptic microRNA/mRNAome is still lacking, and knowledge about other non-coding RNA species is rare. Another problem is that the methods used so far to study synaptic RNAs from tissue samples do not allow us to distinguish between RNAs produced by the corresponding neurons and RNAs that might be transferred to synapses from other cell types. This issue is becoming increasingly important since there is emerging evidence on intercellular RNA transport and data that support the hypothesis that glia cells, for example, provide neurons with RNA [18, 19]. In this study, we isolated synaptosomes from the hippocampus of mice and carried out small RNA sequencing on them. To complement the data and address the question as to the origin of synaptic RNAs, we developed a novel microfluid chamber that not only allowed us to grow primary hippocampal neurons that form synapses in a pre-defined compartment [20] but also enabled us to isolate the synaptic compartments from these chambers using a novel device we call SNIDER (SyNapse Isolation DevicE by Refined Cutting). After the synaptic compartments were isolated, we then performed RNA sequencing. We also show that this novel microfluid chamber is suitable for assaying the dynamics of the synaptic RNAome in response to stimulation.

In conclusion, we were able for the first time to build a high-quality synaptic microRNA/mRNA network, and the data from our experiments point to key synaptic RNAs, including lncRNAs and snoRNAs. These synaptic RNAs will be valuable for future mechanistic studies in the context of the healthy and diseased brain.

## Results

### The Hippocampal Coding and Non-Coding Synaptosomal RNAome

**Fig. 1:**
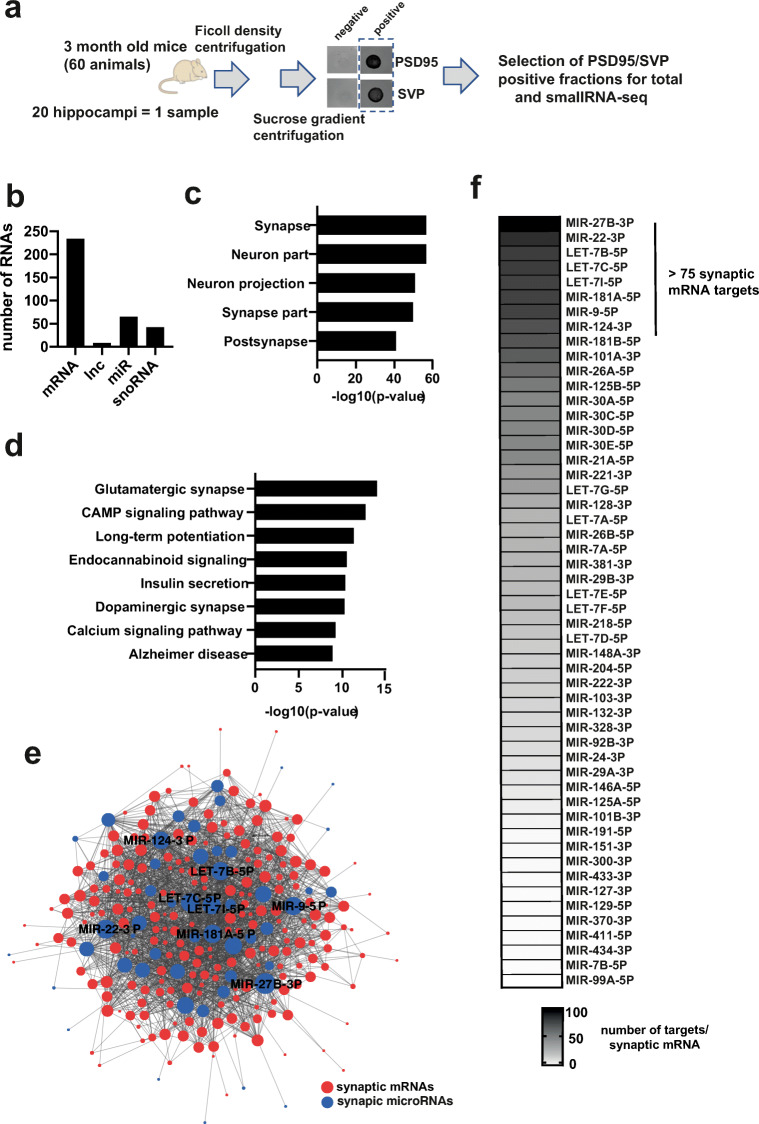
The coding and small non-coding RNAome of hippocampal synaptosomes. **a** Experimental scheme. **b** Bar graph showing the detected RNA species. **c** GO analysis showing that the identified mRNAs represent the synaptic compartment. **d** KEGG-pathway analysis showing that the synaptic mRNAome consists of transcripts that are essential for the function of hippocampal synapses. **e** microRNA-mRNA interaction network of the synaptic RNAome. Red circles represent the identified mRNAs that form a highly connected network, while blue circles indicate the detected microRNAs. Only the names of the top hub microRNAs are shown. **f** Heat map showing the synaptic microRNAome ranked by their confirmed mRNA targets that were found at synapses

We isolated high-quality synaptosomes from the hippocampus of 3-month-old mice and processed the corresponding RNA for total and small RNA sequencing (Fig. 1a). After quality control for high-confidence transcripts, we were able to detect 234 mRNAs, 6 lncRNAs (excluding sequences that code for predicted genes), 65 microRNAs, and 37 SnoRNAs (Fig. 1b; Tables S1, S2, and S3). GO-term analysis revealed that the mRNAs reflect exclusively the pre- and postsynaptic compartments (Fig. 1c), confirming the quality of our data. Functional pathway analysis showed that the mRNAs found in our synaptosomal preparations represent key pathways linked to synaptic function and plasticity (Fig. 1d). We also observed a substantial amount of highly abundant microRNAs in these preparations (Table S2). To understand the synaptic regulatory mRNA-microRNA network, we applied a novel bioinformatic approach which entailed generating the mRNA network using the mRNAs detected at synapses, then intersecting this network with the synaptic microRNAome. We then investigated whether any of mRNAs within the network represented confirmed microRNA targets. Our data revealed that 98% of the synaptic mRNAome was targeted by 95% of the synaptic microRNAs (Fig. 1e, f). This suggests that the synaptic microRNAome plays an important role in local mRNA availability. We detected a number of hub microRNAs, particularly micoRNA-27b-3p, microRNA-22-3p; the cluster consisting of let-7b-5p, let-7c-5p and let-7i-5p; and microRNA-181a-5p, microRNA-9-5p, and microRNA-124-5p. All of these appear to be central regulators of the synaptic mRNA pool (Fig. 1f).

### Comparison of the Hippocampal Synaptosomal Coding and Non-Coding RNAome to Primary Hippocampal Neurons

**Fig. 2:**
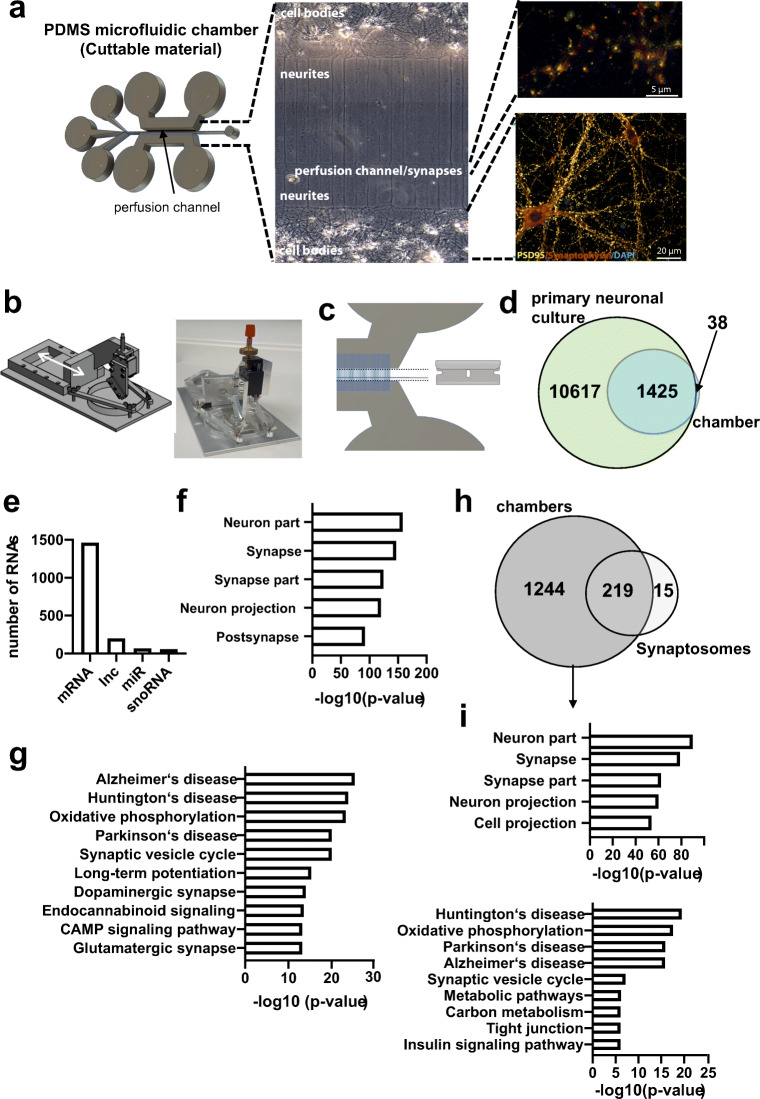
Analyzing the synaptic RNAome in microfluidic chambers via RNA-sequencing. **a** Microfluid chambers built from PDMS. Left panel shows the scheme of the microfluidic chamber indicating the perfusion channel in which most of synapses form. The principle is based on chambers first reported by Taylor and colleagues [20] but has been substantially modified (see Fig S1 for more details). The middle panel shows the bright-field image of neurons growing in these chambers, and the right panel shows immunostaining for PSD-95 and synaptophysin within the perfusion channel (upper image) and the part of the chambers that contains the cell bodies (lower image). **b** Scheme and image showing our newly devised tool for cutting the perfusion channel from the microfluidic chambers, named SNIDER. **c** Schematic illustration of the cutting of the microfluidic chambers. **d** Venn diagram showing the comparison of the total RNA-seq data obtained from primary hippocampal cultures grown in normal dishes (primary neuronal culture) and corresponding data obtained from the perfusion channel isolated from microfluidic chambers in which primary hippocampal neurons were grown. **e** Bar chart showing the detected RNA species. **f** GO analysis showing that the identified mRNAs represent the synaptic compartment. **g** KEGG-pathway analysis showing that the synaptic mRNAome consists of transcripts that are essential for the function of hippocampal synapses. **h** Venn diagram showing the overlap of mRNAs detected in hippocampal synaptosomes and in microfluidic chambers. **i** Upper panel: GO analysis showing that the 1244 mRNAs specifically detected in microfluidic chambers represent the synaptic compartment and “cell projection.” Lower panel**:** KEGG-pathway analysis of the same dataset

Compartmentalized microfluidic chambers have been developed to study the pre- and postsynaptic compartments of neurons. In these chambers, neurons grow their neurites into microgrooves and form synapses in a narrow compartment, the perfusion channel [20]. We hypothesized that such microfluidic chambers would be a *bona fide* complementary approach to study the synaptic RNAome via RNA-sequencing. Moreover, since the synapses formed within the perfusion channel of such chambers are not in contact with any other neural cell type, this approach would also allow us to address the question as to what extent synaptically localized RNAs originate from the corresponding cell or may have been shuttled from neighboring glia cells. This process was proposed for synaptic microRNAs [21]. However, a reliable approach for isolating synapses and corresponding RNA for subsequent sequencing from the perfusion channel of such microfluidic chambers did not exist at that time. Therefore, we generated a modified microfluidic chamber that allowed us to cut the perfusion channel to harbor the corresponding synapses, after which we performed the isolation of RNA. Mouse hippocampal neurons were thus grown in these chambers (Fig. 2a). For reproducible cutting, we employed a newly devised instrument we call SNIDER (SyNapse Isolation DevicE by Refined Cutting) (Fig. 2b, c) and isolated RNA for total and small RNA sequencing. When comparing the transcriptome obtained from the perfusion channel with corresponding data generated from RNA isolated from primary hippocampal neurons grown in normal culture dishes, we observed the expected enrichment for a specific subset of RNAs, representing about 12% of the entire transcriptome (Fig. 2d). In more detail, the transcriptome of the perfusion chamber consisted of 1460 mRNAs, 199 lncRNAs, 54 microRNAs and 57 highly expressed snoRNAs, of which 22 were also detected in synaptosomes. (Fig. 2e; Supplemental tables S4, S5, S6). GO-term analysis revealed that the identified mRNAs represent the synaptic compartment, which is in line with our data obtained from the adult mouse hippocampus (Fig. 2f) and further supports the feasibility of our approach. Functional pathway analysis confirmed that the detected mRNAs code for key synaptic pathways and reflect the high energy demand of synapses (oxidative phosphorylation). This is also the reason why pathways such as Alzheimer’s, Huntington’s, and Parkinson’s disease are identified (Fig. 2g), since key genes deregulated in these diseases are linked to mitochondria function. The direct comparison of the hippocampal synaptic mRNAome from the adult mouse brain and the mRNAome from primary neurons revealed that almost all mRNAs detected from *in vivo* synaptosomes are also found in primary neurons grown in microfluidic chambers (Fig. 2h), confirming 219 mRNAs as a high-quality and reproducible synaptic mRNAome. The GO terms and functional pathways linked to these 219 mRNAs are identical to the data shown in Fig. 1 c and d. The 1244 mRNAs that were specifically observed in microfluidic chambers also represent the synaptic compartments and pathways linked to oxidative phosphorylation, synaptic vesicle cycle, and metabolic processes and may therefore reflect the difference between the synaptic RNAome in the adult brain and in cultured primary neurons (Fig. 2i). In addition, “neuronal projection” was detected as a significant GO term, most likely indicating the fact that in contrast to synaptosomal preparations, the perfusion channel still contains some neurites. This might also explain why many more lncRNA, in particular 199-annotated lncRNAs, are detected in the microfluidic chambers. Pathway analysis suggests that these lncRNA are mainly linked to mRNAs that control processes associated with oxidative phosphorylation and synaptic plasticity, while comparatively few microRNAs seem to be regulated by the synaptic lncRNAs (Fig. S2). Similar to the *in vivo* data, we found 54 highly expressed microRNAs (Table S5).

**Fig. 3:**
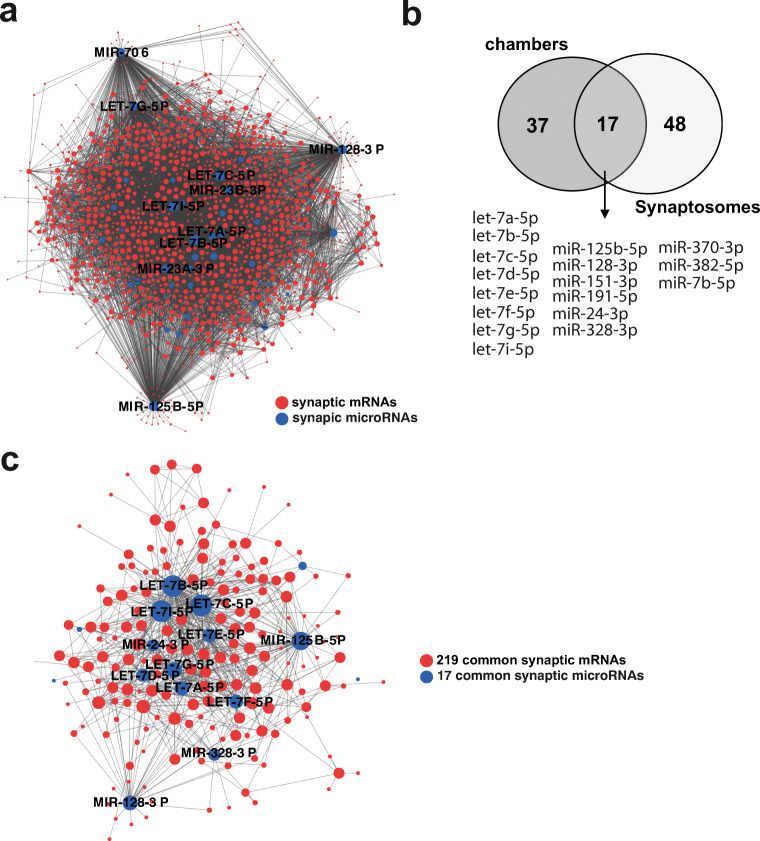
A core synaptic microRNAome. **a** MicroRNA-mRNA interaction network of the synaptic RNAome detected in microfluidic chambers. Red circles represent the identified mRNAs that form a highly connected network, while blue circles indicate the detected microRNAs that control this network. Only the names of the top hub microRNAs are shown. **b** Venn diagram comparing microRNAs detected in microfluidic chambers (Chambers) and synaptosomes. **c** MicroRNA-mRNA interaction network of the 219 synaptic mRNAs commonly detected in synaptosomes and microfluidic chambers and the 17 commonly detected microRNAs. Only the names of the top hub microRNAs are shown

To further study the mRNA/microRNA network, we used the same approach as described for the synaptosomal data. Our data reveals that 88% of the synaptic mRNAome in microfluidic chambers is targeted by 45 (83%) synaptic microRNAs (Fig. 3a). Taken together, our data from hippocampal synaptosomes and the novel microfluidic chamber strongly suggest that the synaptic transcriptome is under tight control of a local microRNA network. Comparison of the *in vivo* synaptic microRNAome with the data obtained from the microfluidic chambers revealed 17 microRNAs that were commonly identified at synapses, while 37 microRNAs were specific to the chambers and 48 microRNAs were only found in the *in vivo* data from hippocampal synaptosomes (Fig. 3b). When we generated the synaptic microRNA/mRNA network for the commonly detected 17 synaptic microRNAs and 219 mRNAs (see Fig. 2g), we observed that this core synaptic microRNAome controls 80% (179 of 219) of the core mRNAome (Fig. 3c).

### Evidence for Astrocytic microRNA Transport to Synapses

**Fig. 4:**
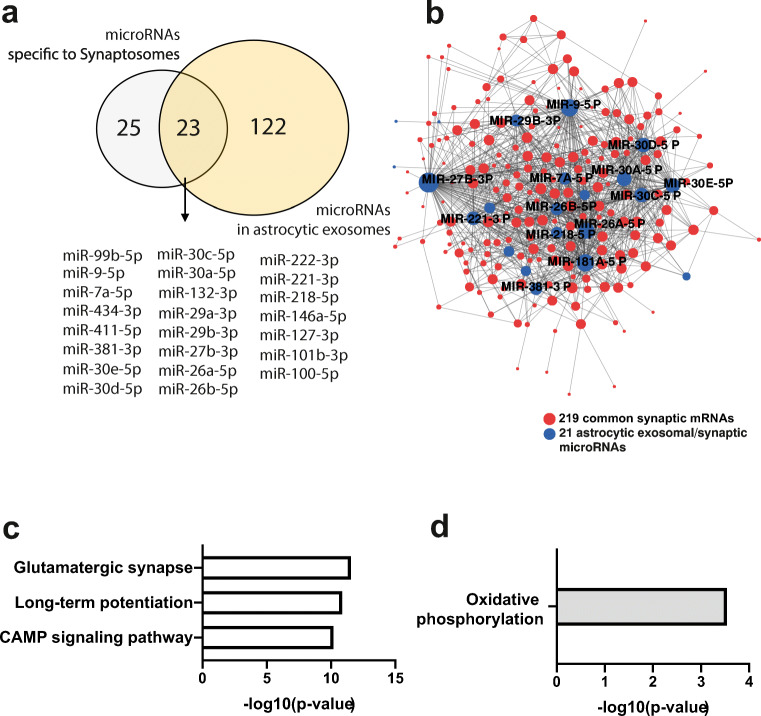
Comparing microRNAs from astrocytic exosomes to the synaptic RNAome. **a** Venn diagram comparing the 48 microRNAs exclusively detected in synaptosomes to the list of microRNAs found in astrocytic exosomes. **b** MicroRNA-mRNA interaction network showing that 203 of the commonly detected 219 mRNAs and 21 of the 23 microRNAs found in synaptosomes and astrocytic exosomes form an interaction network. Only the names of the top hub microRNAs are shown. **c** KEGG-pathway analysis of the 203 mRNAs within the network. **d** KEGG pathway analysis of the 16 common synaptic mRNAs that are not targeted by the overlapping microRNAs shown in **a**

We found 37 microRNAs exclusively in synapses from primary neurons. This is most likely due to the difference between *in vivo* brain tissue and primary neuronal cultures, and a similar trend has been observed at the level of the mRNAs (see Fig. 2g). More interesting is the observation that 73%, namely, 48 out 65, of the microRNAs detected in hippocampal synaptosomes were not found in microfluidic chambers (see Fig. 3b), which is in contrast to the mRNA data in which almost all of the synaptosomal mRNAs were detected in synapses of the primary neuronal cultures grown in microfluidic chambers (see Fig. 2g). These data may indicate that *in vivo*, some of the synaptic microRNAs are not exclusively produced by the corresponding neuron but rather may be shuttled to synapses via other neural cell types. In fact, movement of microRNAs between cells is an accepted mechanism of intracellular communication [18]. Prime candidate cells to support synapses with microRNAs are astrocytes that form together with neurons tripartite synapses. A prominent mechanism that mediates RNA transport amongst neuronal cells is intracellular transport via exosomes [22]. Thus, we compared a previously published dataset in which microRNAs from astrocytic exosomes were analyzed via a TAQman microRNA-array [23]. Indeed, 50% of the microRNAs exclusively detected in hippocampal synaptosomes have also been described in exosomes released from astrocytes (Fig. 4a). When we examined whether these 23 microRNAs had mRNA targets in synaptosomes, we observed that 21 of these microRNAs targeted a total of 197 of the 219 commonly detected synaptic RNAs (Fig. 4b). This was further confirmed by functional pathway analysis showing that the 21 microRNAs control synaptic genes linked to the glutaminergic synapse, LTP, and cAMP signaling (Fig. 4c). It is interesting to note that the synaptic mRNAs not targeted by any of the 21 microRNAs represented functional pathways linked to oxidative phosphorylation (Fig. 4d).

### Synaptic MicroRNAs are Linked to Neurodegenerative and Neuropsychiatric Diseases

So far, our data support the view that the synaptic microRNAome plays an important role in neuronal function. To further strengthen this notion, we decided to examine whether synaptic microRNAs are particularly deregulated in cognitive diseases. To this end, we performed a literature search and curated a list of 71 microRNAs that were found to be deregulated in post-mortem human brain tissue, blood samples, or model systems for Alzheimer’s disease, depression, bipolar disease, or schizophrenia. Comparison of this dataset with our findings from synaptosomes revealed 17 synaptic microRNAs that are deregulated during cognitive diseases, four of which were also found in the microfluidic chambers and 11 in astrocytic exosomes. These microRNAs thus provide an interesting pool of synaptic microRNAs for further studies (Table S1).

### Microfluidic Chambers are Suitable to Assay the Synaptic RNAome upon Neuronal Stimulation

**Fig. 5:**
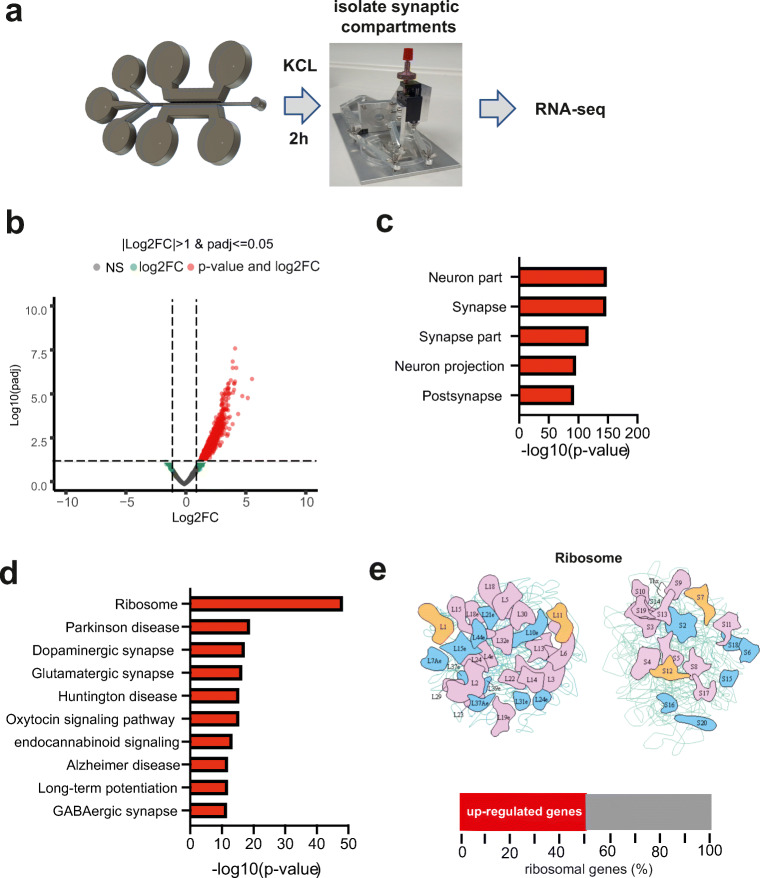
The synaptic mRNAome upon stimulation. **a** Experimental scheme. **b** Volcano plot showing a substantial upregulation of synaptic RNAs upon KCL treatment. **c** GO analysis showing that the identified mRNAs represent the synaptic compartment. **d** KEGG pathway analysis showing that the changes of the synaptic mRNAome upon KCL treatment represent transcripts mainly linked to ribosomal function. **e** Upper panel shows images of the KEGG pathway for “ribosome.” Colored subunits represent transcripts significantly increased. Lower panel: bar chart showing that 50% of the genes that comprise the ribosome KEGG pathway are increased at the synaptic compartment upon KCL treatment

Our findings suggest that the synaptic RNAome can be studied in a reliable manner using our modified microfluid chambers in combination with SNIDER. This approach also provides a novel tool to investigate the neuronal-controlled synaptic RNAome in response to stimulation. To further evaluate this potential, we decided to expose primary hippocampal neurons grown in microfluidic chambers to KCl treatment and then isolate the perfusion channel 2 h later. The isolated RNA was used for RNA sequencing (Fig. 5a). Our analysis revealed a substantial number of mRNAs that were increased in the synaptic compartment (Fig. 5b; Table S7). Since we can exclude that these mRNAs are shuttled from glia cells, they likely represent part of the transcriptional response and reflect mRNAs that were transported to synapses, which is feasible within the 2-h time window after treatment. In line with this assumption, the upregulated RNAs exclusively represent the synaptic compartment (Fig. 5c). Functional pathway analysis revealed a strong enrichment of RNAs coding for the ribosome (Fig. 5d). In fact, 50% of all transcripts that corresponded to the ribosomal subunits were increased at the synapse upon KCL treatment (Fig. 5e).

## Discussion

### The Synaptic RNAome

The aim of our study was to provide a high-quality dataset of the synaptic coding and small non-coding RNAome with a specific focus on microRNAs. Our findings thus represent an important resource for future studies. To the best of our knowledge, our study also provides the first dataset which analyzes in parallel the coding, non-coding, and small non-coding RNAome in hippocampal synapses via next-generation sequencing. Moreover, we used two different approaches: we isolated hippocampal synaptosomes from the hippocampus of 3-month-old wild-type mice, and we developed a microfluidic chamber that in combination with a novel cutting device allowed us to isolate synaptic compartments for subsequent RNA sequencing from primary hippocampal neurons. This chamber combines the advantages of the currently used microfluidic chambers that allow the specific manipulation of synapses [20] with the ability to isolate the perfusion channel that harbors synaptic connections. Therefore, this novel microfluidic chamber will facilitate the specific manipulation of the synaptic compartment in combination with next-generation sequencing approaches. It should thus be considered a suitable screening tool to study the dynamics of the synaptic RNAome. The feasibility of this approach was demonstrated for example by our finding that KCL treatment leads to substantial changes in the synaptic RNAome. Future approaches will now be able to employ more physiological manipulations and thus investigate the synaptic RNAome in disease models. It is noteworthy that most of the mRNAs upregulated at the synapse upon stimulation represent key components of the ribosome, which is in agreement with the importance of local mRNA translation [4].

In line with previous data, we identified a substantial number of mRNAs that almost exclusively represent the synaptic compartment and key signaling pathways linked to synaptic integrity and plasticity. It is interesting that the mRNA coding for the amyloid precursor protein (APP), a key factor in Alzheimer’s disease (AD) pathogenesis, was also found at synapses (see Table S1). To our knowledge, this observation has not been reported before but is in line with the physiological function of wild-type APP at synapses [24].

Generally, we detected more mRNAs within the dataset obtained from primary neurons when compared with the synaptosomal preparation. This observation likely reflects the difference between the *in vivo* preparation of hippocampal tissue and cultured primary neurons. Another important consideration is that synapses likely differ depending on the distance to the soma, an issue that cannot be addressed when isolating synaptosomes, while the RNAome detected in the microfluidic chambers represents synapses that are most distant to the corresponding somata. Similarly important is the fact that the preparation from the perfusion channel of our microfluid chamber still contained some neurites. Thus, the corresponding RNAome also included dendritic mRNAs. This view is supported by previous data in which the mRNA pool was analyzed from neuropil or synapto-dendritic compartments. For example, 2550 mRNAs were detected in hippocampal neuropil from mice [8], and 1875 mRNAs were identified when ribosome-bound mRNA was analyzed in the same region [7]. We observed only 234 mRNAs in hippocampal synaptosomes, but we consider these mRNAs to represent a high-quality dataset. Thus, we only report here mRNAs that passed rigorous quality control and exhibited a substantial amount of sequencing reads. The quality of these data is further confirmed by the fact that almost all of the synaptosomal mRNAs, namely 219, were also detected in the RNA-seq dataset we obtained from the microfluidic chambers. The most comparable mRNA dataset with our *in vivo* approach is a recent study that employed FACS to isolate synaptosomes from the mouse forebrain [12] and also reported raw data on the generic synaptosomes. It is important to note that the latter study employed a different analysis pipeline than we did and reported all transcripts that map with > 25% of the read length when using the STAR-aligner tool, while we considered only transcripts that map with at least >66%. Nevertheless, we observed that the top 500 mRNAs reported by Hafner et al. almost completely overlapped with our dataset. Namely, 209 of the 234 mRNAs that we reported for hippocampal synaptosomes are also found in the Hafner et al. dataset from mouse forebrain synaptosomes (Table S8), further supporting the quality of our dataset and strengthening the view that synaptic mRNAs play a critical role in neuronal function. We also report the detection of lncRNAs in datasets obtained from synaptosomes and microfluidic chambers but for now restricted the presented data to the currently annotated lncRNAs. We also identified lncRNAs that are currently still referred to as “predicted” and awaiting further confirmation. Therefore, we encourage researchers to further explore our raw data since annotation of the genome is improving. The presence of lncRNA in synaptosomes is in line with previous data [11], but it is interesting to note that more lncRNAs were found in the microfluidic chambers when compared with the *in vivo* synaptosomes. A similar trend has been observed for mRNAs and might be due to the fact that the RNA preparation from the microfluid chambers also contains some dendritic RNA. Our data suggest that the detected lncRNAs regulate processes associated with oxidative phosphorylation and synaptic plasticity and may also affect the function of selected microRNAs. Although these observations need to be further studied, it is interesting to note that metastasis-associated lung adenocarcinoma transcript 1 (Malat1) appeared as one hub lncRNA at synapses. This is in line with a previous study showing that knocking down MALAT1 in hippocampal neurons decreases the number of synapses, although it must be mentioned that the authors linked this finding to the role of MALAT1 on gene expression control [25]. The presence of snoRNAs at synapses is also highly interesting and corresponds with a previous study that reported snoRNAs in synaptosomes [26]. Moreover, there was a substantial overlap of the snoRNAs detected in synaptosomes and in primary neurons (60% of the synaptosomal snoRNAs were also detected in microfluidic chambers). Most of the commonly detected snoRNAs were of the C/D box (49%) or H/ACA-box type (17%) that regulate RNA-methylation and pseudo-uridylation of mainly ribosomal RNAs [27], which further underlines the presence of ribosomes at synapses [4]. However, we also identified snoRNAs that cannot be classified in either category (35%); these warrant further investigation. Some of the synaptic snoRNAs have been associated with additional processes, for instance SNORD50, SNORD83B, or SNOR27 which have been linked to mRNA 3′ processing and post-transcriptional gene-silencing [27]. SNORD115, however, affects mRNA abundance and is genetically linked to the Prader-Willi syndrome, a rare genetic disease that leads to intellectual disability [28].

### A synaptic mRNA/microRNA Network

We detected a substantial number of microRNAs in hippocampal synaptosomes and in the microfluidic chambers. The presence of mature microRNAs at synapses is in line with previous reports that employed RT-PCR to study neurites of primary hippocampal neurons [29], microarray technology to analyze microRNAs in the synapto-neurosomes isolated from the forebrain of mice [30], or more recently also small RNA sequencing and NanoString analysis of hippocampal neuropil or synaptosomes [15, 26]. Comparison of the dataset generated by Sambandan and colleagues revealed that out of the 65 microRNAs we detected, 57 were also reported in their study. This further strengthens the hypothesis that microRNAs play an important role at synapses and suggests that our dataset represents a high-quality synaptic microRNAome that can be used as a resource for future studies. To the best of our knowledge, our study is the first that provides a synaptic coding and small non-coding RNAome from the same preparation, thus making it possible to address the role of the synaptic microRNAome at the systems level. We used the data to develop a novel tool which utilized mRNA data to parse multiple databases containing experimentally validated interactions and thereby build a high-confidence mRNA network of the synapse (see methods for more details). We intersected this mRNA network with the confirmed targets of all microRNAs which are detected within the same sample to build the synaptic microRNA/mRNA network. Overall, our data suggest that up to 98% of the synaptic mRNAome is controlled by synaptic microRNAs, suggesting that essentially, all mRNAs localized at the synapses are potentially regulated via synaptic microRNAs. Since mRNA transport to synapses is an energy-demanding and highly controlled process [2], it is likely that synaptic microRNAs do not degrade their mRNA targets but rather control their availability for local translation. This should be studied in future experiments at the systems level. Another important observation was that many of the synaptic microRNAs were deregulated in cognitive diseases that often start with synaptic dysfunction (see Table S1). In addition, there is increasing interest in circulating microRNAs as biomarkers for cognitive diseases [31, 32]. The fact that microRNAs have also been reported in synaptic vesicles [33] and in exosomes derived from neuronal cultures [34] suggests a potential path how pathological microRNA changes observed in the brain may also manifest in circulation. Hence, the various CNS clearance systems [35] might transport such vesicles to the circulation, a hypothesis that should be further studied. In the same context, there is substantial data to suggest that microRNAs regulate biological processes across cell types and even organs [18, 36]. Intriguingly, in the perfusion channel of microfluidic chambers—which are free of any somata and only contain distal synapses and some neurites—there are substantially fewer microRNAs than in the synaptosomes. These microRNAs significantly overlapped with the ones detected in exosomes released by astrocytes [23]. It is therefore tempting to speculate that within the tripartite synapse, astrocytes support synapses with additional microRNAs that help to control the synaptic mRNA pool. Support for this view stems also from the observation that the three most significant functional pathways controlled by the synaptosomal microRNAome are the “glutamatergic synapse,” “cAMP signaling,” and “long-term potentiation,” which are identical to the top three pathways controlled by the microRNAs that are potentially shuttled to synapses via astrocytes. These data underscore the importance of the corresponding mRNA pool and may suggest that microRNAs supplied to synapses by other cell types might suppress translation of the most relevant local mRNAs rather than degrading a few selected RNAs. Our data allowed us to identify a number of synaptic hub microRNAs (e.g., see Fig. 1 e and f), and the functional analysis of these microRNAs would be an important task for future studies. Of particular importance would be microRNAs that are deregulated in cognitive diseases. Support for this view stems from recent data on microRNA-181a-5p, a hub in our synaptic network that is deregulated in neurodegenerative and neuropsychiatric diseases [37, 38] and was found to be processed at synapses upon neuronal activity [15]. The finding that most microRNAs of the let-7 family are highly abundant at synapses and control a large set of mRNAs is intriguing since these microRNAs have been observed in several CNS-related pathologies [39] while comparatively little is known on their role in the adult brain. Another hub microRNAs is miR-125b-5p which is deregulated in Alzheimer’s disease and causes memory impairment in mice when elevated in their hippocampus [40], yet its role at the synapse remains elusive. Similarly interesting is miR-128-3p which is deregulated in various neuropsychiatric and neurodegenerative diseases. Recent data suggest that inhibition of microRNA-128-3p can ameliorate AD pathology [41].

In conclusion, our study provides for the first time the synaptic RNAome and thus will serve as a valuable resource for future research. Our data furthermore underscore the importance of synaptic mRNAs and microRNAs for brain function. The novel microfluidic chamber that we developed here will allow researchers to combine the power of a specific analysis and manipulation of the synaptic compartment [20] with RNA-sequencing approaches.

## Materials and Methods

### Animals

Three-month-old male C57B/6J mice were purchased from Janvier Labs. All animals were housed in standard cages on 12-h/12-h light/dark cycle with food and water ad libitum. All experiments were performed according to the protocols approved by the local ethics committee of the University Medical Center of the University Goettingen, Germany, the Lower Saxony State Office for Consumer Protection and Food Safety (LAVES) under animal protocol number T12.13 and were in compliance with institutional, national, and international guidelines. The 3Rs (replacement, reduction and refinement) were applied in all animal research activities.

### Isolation of Hippocampal Synaptosomes for RNA Sequencing

To obtain sufficient RNA for sequencing of hippocampal synaptosomes, we isolated the hippocampi from 60 3-month-old wild-type mice. Twenty bilateral hippocampi were pooled as one sample to obtain three independent samples that were further processed to isolate high-quality synaptosomes using a previously described protocol [42]. In brief, hippocampi were homogenized by 9 strokes at 900 rpm in sucrose buffer and centrifuged at 4° for 2 min at 5000 rpm (SS34). Supernatants were further centrifuged at 4° for 12 min at 11,000 rpm. Pellets were loaded onto a Ficoll gradient and centrifuged at 4° for 35 min at 22,500 rpm (SW41). The interface between 13 and 9% Ficoll was washed by further centrifugation and then pelleted by 8700 rpm for 12 min in a SS34 rotor. Resuspended synaptosomes were then centrifuged on a sucrose gradient for 3 h at 28,000 rpm (SW28). Finally, synaptosomes were fractioned via the Gilson Minipuls, and 21 fractions were collected and analyzed by dot blotting. For this, from each fraction, 2 μl of sample was pipetted onto nitrocellulose membrane, and dried for 5 min. Blocking of unspecific signal was done by 5% low-fat milk in TBST for 10 min. Antibodies against synaptophysin and PSD95 were applied for 15 min, then the membrane was washed three times for 3 min each in TBST with 5% milk. Secondary antibody was applied for 15 min. Afterward, membrane was washed again three times with TBST without milk before being imaged. Only 5 fractions from each preparation showed a signal for synaptophysin and PSD95, ensuring the presence of high-quality synaptosomes. These were then processed for total and small RNA sequencing.

### Production of Microfluidic Chambers

Isolation of synapses and corresponding RNA for subsequent sequencing from the perfusion channel of currently employed microfluidic chambers [20] was not possible. Therefore, we generated a microfluidic chamber that allowed us to cut the perfusion channel by using polydimethylsiloxane (PDMS) for the chamber and the corresponding substrate (Fig S1). Pilot studies showed that unlike the commonly used microfluidic chambers [20], the usage of PDMS as a substrate to bind the chambers allowed us to cut the perfusion channel. Specifically, the microfluidic chambers were designed using AutoCAD 2017. The overall layout was similar to the version reported by Taylor and colleagues [20], yet for more yield of synaptic RNAs, the length of the chamber was increased, with more microgrooves and a wider synaptic compartment to allow easier alignment during cutting. Layouts were translated into photolithography masks by Selba. Production of silicon wafers was done with two layers. The first layer was made by applying 2 ml photoresist SU-8-2025 on 2 inches-diameter silicon wafers and running the spin coater with the following settings: (1) 15 s, 500 rpm, 100 ramp and (2) 100 s, 4000 rpm, 50 ramp. To prebake, the wafers were put on a 65°C heating plate for 1 min, then for 15 min on a 95° heating plate. For depositing the first layer to UV light, the mask with the microgrooves pattern was inserted into the MJB4 mask aligner; exposure was set to 9 s under vacuum conditions. Afterward, the wafers were postbaked at 65° for 1 min and 5 min at 95°C.

Subsequently, 3 ml of the second photoresist SU-8-2050 was added on top and spin coated with the following protocol: (1) 15 s, 100 ramp, 500 rpm and (2) 60 s, 900 rpm, 50 ramp. This time, prebaking was done with 1 min at 65° and a minimum of 30 min at 95°. The second layer was aligned to the microgrooves using the microscope of the mask aligner. UV light exposure lasted 19 s in the soft contact setting. After postbaking as described for the first layer, wafers were developed for 10 min or more in mrDev600 with the aid of ultrasonication. PDMS (SYLGARD™ 184 Silicone Elastomer Kit) was used to manufacture the chambers as well as the bottom substrates. Sylgard components were mixed 10:1 using a 1-ml pipette tip and added to the wafers that were placed in 6-cm-diameter Petri dishes and very thinly (1–2 mm high) onto 10-cm dishes. Degassing was performed for minimum 15 min in a desiccator under vacuum. Afterward, wafers and bottom parts were transferred to a 70° oven and cured for 2 h. Chambers and bottom parts were cut out by a scalpel, holes in the chambers were punched by biopsy punchers of 6 mm and 8 mm diameter, and the bottom parts were cut into smaller pieces to hold one chamber each. To clean off dust, the pieces were placed in an ultrasonic bath for 10 min and then dried on a heating plate at 70°. PDMS can be bound to PDMS covalently under oxygen plasma conditions; a Tesla-coil type device, the Corona plasma treater from Blackhole lab, was used to this end. The plasma treater was hovered slowly 2 cm above the chambers (bottom side up), going back and forth to cover the entire area by discharges for 30 s, then the same was done to the bottom part. Both parts were then brought together and pressed very slightly to ensure complete contact. Covalent bond forming was enhanced by placing the assembled chamber into the oven at 70° for 10 min. Subsequently, chambers were filled with PBS or borate buffer to maintain hydrophilic properties. Chambers that were to be imaged were not treated with plasma but were assembled onto the PDMS or glass substrate under the biosafety cabinet by simply pressing both pieces together. Once assembled, the chambers were brought to a biosafety cabinet and washed once with 70% ethanol, then twice with water. Coating on PDMS worked best when done with 0.5 mg PDL in borate buffer overnight. Visual inspection under the microscope ensured that no bubbles were present in the chambers. Great care was taken when washing to not remove the coating. Liquid was never removed with a suction pump directly from the channels but rather by pointing the pipette at the wall of open reservoirs. Washing was done twice with PBS, 80 μl per top reservoir, allowing for the liquid to flow into the down reservoir. Perfusion reservoirs were washed by applying 50 μl onto each well, one at a time, waiting for 5min in-between. Once all PBS was removed from the open reservoirs, 80 μl of medium was added per top reservoir, allowing for the liquid to flow into the down reservoir. This process was repeated once before the chambers were left overnight in the incubator before seeding to ensure proper hydrophilicity. For easier handling, always two chambers were put together in a 10-cm dish, with two lids of 15-ml falcon tubes filled with water next to them to reduce the evaporation from the chambers themselves.

### Primary Hippocampal Neuronal Cultures

Pregnant CD1 mice were sacrificed under anesthesia by cervical dislocation at E16 or E17. Brains from embryos were extracted and their hippocampi collected. Processing was done using the Papain kit from Worthington, and the cells were counted and diluted to a density of 5 million per ml. Seeding was done with the following pipetting scheme to ensure that most cells reached the microgrooves but did not enter them: 10μl of cell suspension containing 70,000 cells was injected into the channels from the top wells, starting with the axonal side. A second pipetting step with 5 μl added to the channels from the bottom wells, after inspection of cells under the microscope. After 10 min, a similar seeding was performed for the dendritic side. One hour later, each well was filled up to 100 μl. The next day, another 100 μl was pipetted into each well. Visual inspection under a microscope was necessary to do several rounds of seeding with decreasing volume to make sure the desired spread of cells was achieved. After 2 h, reservoirs of the chambers were filled up with medium to 100 μl each by pipetting an additional 70 μl simultaneously in both reservoirs per side, while not adding more medium to the perfusion. We used Neurobasal Plus with GlutaMax, Penicilin/Strep, and B27 Plus supplement for better viability. Parallel to chambers, normal 12-well dishes, coated with PDL in borate overnight and washed three times with water, were cultured at 260,000 cells per well; these served as standby cultures. Since medium evaporation can happen quickly in the chambers, every 2–3 days, medium from these standby cultures was filtered by a 0.22-μm syringe filter and then added to the chambers. For the KCl stimulation, around 50 μl of medium was collected from each reservoir of the chambers and mixed with KCl, resulting in a final concentration of 50 mM when given back to the chamber and then incubated for 2 h before RNA isolation.

### Harvesting of Synaptic RNAs from Microfluidic Chambers: SNIDER

In order to parallel cut the PDMS substrate, we designed a machine consisting of a blade-holding arm on a ball-bearing rail, allowing frictionless mobility in one dimension. A screw-driven spring drives the razorblade height position and allows for controlling the penetration depth of the blades into the PDMS. The non-cutting corners of the razorblade were removed with a plunger to only have one accessing point of the blades into the PDMS. Small metal plates were put in between the blades to serve as spacers, increasing the inter-blade distance to 900 μm. On the day of harvest, cells in the chambers were washed once with PDMS and flipped upside down. Great care was taken to maintain an RNAse-free environment by prior cleaning of all tools and instruments with RNAsezap and 70% EtOH afterward. To obtain an endpoint for the long parallel cut, we introduced with a scalpel two horizontal cuts between the outer perfusion wells and the upper left, respectively upper right well that met at the perfusion stream. Then, the chambers were aligned by their perfusion stream on a marked line of the device. By close visual inspection, the blades were lowered just before entering the PDMS material and then were brought in parallel to the synaptic compartment. Blades were then lowered 2 mm deep into the substrate just before the perfusion outlet and then the metal lever was pulled backward, moving the blades toward the perfusion wells until the parallel cut met the V-shaped cut induced earlier by the scalpel. With a pair of tweezers, the synaptic compartment was taken out and put into cell lysis buffer solution of GenElute Sigma kit. We then followed the manufacturer’s protocol under 1C to isolate total RNA, including small RNAs.

### RNA Sequencing

The synaptosomal RNA samples were split into halves; one was further processed to obtain total RNA libraries using the Illumina Truseq total RNA kit, and the other half was used for small RNA sequencing using the NEBNext Small RNA Library Prep Kit as described before [43]. For total RNA sequencing of RNA from microfluid chambers, we always pooled two samples, and libraries were created with Takara´s SMARTer Stranded Total RNA-Seq Kit v2-Pico Input Mammalian. Small RNA libraries were generated using Takara’s SMARTer smRNA-Seq Kit for Illumina. To verify the library and sequencing procedure, we added spike-in RNAs from the QIAseq miRNA Library QC kit prior to library creation.

### Bioinformatic Analysis

Sequencing data was processed using a customized in-house software pipeline. Illumina’s conversion software bcl2fastq (v2.20.2) was used for adapter trimming and converting the base calls in the per-cycle BCL files to the per-read FASTQ format from raw images. Quality control of raw sequencing data was performed by using FastQC (v0.11.5). Trimming of 3′ adapters for small RNASeq data was done using Cutadapt (v1.11.0) (10.14806/ej.17.1.200). The mouse genome version mm10 was used for alignment and annotation of coding and non-coding genes. Small RNAs were annotated using miRBase [44] for miRNAs and snOPY [45] for snoRNAs. For total RNASeq, reads were aligned using the STAR aligner (v2.5.2b) [46], and read counts were generated using feature Counts (v1.5.1) [47]. For small RNASeq, reads were aligned using the mapper.pl script from mirdeep2 (v2.0.1.2) [48] which uses bowtie (v1.1.2) [49], and read counts were generated with the quantifier.pl script from mirdeep2. All read counts were normalized according to library size to transcript per million (TPM). We used a TPM cutoff of 1000 reads for small RNAs to make sure that these were effectively detected up to an average raw count of 10 reads. To account for differences in sequencing depth between synaptosomal mRNAs (average of 6 mio unique reads per lane) and mRNAs from microfluidic chambers (average of 20 mio unique reads per lane), we applied a cutoff of 50 and 100 normalized reads, respectively. Differential expression analysis was performed with the DESeq2 (v1.26.0) R (v3.6.3) package [50]; here, unwanted variance was removed using RUVSeq (v1.20.0) [51]. Networks were built using Cytoscape (v3.7.2) [52] based on automatically created lists of pairwise interactors. We used in-house Python scripts to detect interactions between expressed non-coding RNAs (miRNAs, lncRNAs, or snoRNAs) and coding genes; interaction information was collected from six different databases: NPInter [53], RegNetwork [54], Rise [55], STRING [56], TarBase [57], and TransmiR [58]. All interactions classified as weak (if available) were excluded. The lists of pairwise interactors were loaded into Cytoscape, and all nodes connected by only one edge were removed to build the final network.

### Imaging

Cells were fixed in 4% PFA in PBS plus 1 μM MgCl2, 0.1 μM CaCl2, and 120 mM sucrose. Our imaging setup consists of a Leica DMi8 microscope that is equipped with a STEDYcon. Phase contrast images were obtained using the Leica in its normal mode, with the Leica DMi8 software. All other fluorescent images were taken with the STEDYcon in either confocal or STED mode. Antibodies: PSD95 (Merck - MABN 68) and synaptophysin 1 (Synaptic Systems 101 004), both diluted to 1:400. Secondary antibodies were StarRED (Abberior, STRED-1001-500UG) and Alexa Fluor 633 Anti-Guinea Pig (Invitrogen, A21105) both diluted to 1:400. DAPI was applied for 1 min for counterstaining.

## Supplementary information

ESM 1 (PDF 5778 kb)

ESM 2 (XLSX 83 kb)

## Data Availability

All sequencing data are available via GEO database. GSE159248: https://www.ncbi.nlm.nih.gov/geo/query/acc.cgi?acc=GSE159248

## Declarations

This work includes experiments with mice. All described experiments were approved by the local animal care committee.
