# Correction to: The Coding and Small Non-coding Hippocampal Synaptic RNAome

**DOI:** 10.1007/s12035-021-02349-2

**Published:** 2021-03-12

**Authors:** Robert Epple, Dennis Krüger, Tea Berulava, Gerrit Brehm, Momchil Ninov, Rezaul Islam, Sarah Köster, Andre Fischer

**Affiliations:** 1 Department of Systems Medicine and Epigenetics, German Center for Neurodegenerative Diseases (DZNE), Von Siebold Str. 3a, 37075 Goettingen, Germany; 2 Bioinformatics Unit, German Center for Neurodegenerative Diseases (DZNE), Von Siebold Str. 3a, 37075 Goettingen, Germany; 3 Institute for X-Ray Physics, University of Goettingen, Goettingen, Germany; 4 Cluster of Excellence “Multiscale Bioimaging: from Molecular Machines to Networks of Excitable Cells” (MBExC), University of Goettingen, Goettingen, Germany; 5 Department of Neurobiology, Max-Planck Institute for Biophysical Chemistry, 37077 Göttingen, Germany; 6 Department of Psychiatry and Psychotherapy, University Medical Center Goettingen, Goettingen, Germany

**Correction to: Molecular Neurobiology**

10.1007/s12035-021-02296-y

The original version of this article unfortunately contained some mistakes.

There are changes in the authorship order and the list of authors. Dr. Momchil Ninov is added as the fifth author.

